# Academic and Behavioral Outcomes in School-Age South African Children Following Severe Traumatic Brain Injury

**DOI:** 10.3389/fnana.2017.00121

**Published:** 2017-12-13

**Authors:** Aimee K. Dollman, Anthony A. Figaji, Leigh E. Schrieff-Elson

**Affiliations:** ^1^Applied Cognitive Science and Experimental Neuropsychology Team, Department of Psychology, University of Cape Town, Cape Town, South Africa; ^2^Division of Neurosurgery, Department of Surgery, School of Child and Adolescent Health, University of Cape Town, Red Cross War Memorial Children's Hospital, Cape Town, South Africa

**Keywords:** pediatric, traumatic brain injury, academic, behavior, outcome, developing countries

## Abstract

**Background:** Children who have sustained severe traumatic brain injuries (TBIs) demonstrate a range of post-injury neurocognitive and behavioral sequelae, which may have adverse effects on their academic and behavioral outcomes and interfere with school re-entry, educational progress, and quality of life. These post-TBI sequelae are exacerbated within the context of a resource-poor country like South Africa (SA) where the education system is in a somewhat precarious state especially for those from disadvantaged backgrounds.

**Objectives:** To describe behavioral and academic outcomes of a group of school-aged SA children following severe TBI.

**Methods:** The sample included 27 school-age children who were admitted to the Red Cross War Memorial Children's Hospital (RXH), SA, between 2006 and 2011 for closed severe TBI and who received intracranial monitoring. We collected behavioral data using the Child Behavior Checklist (CBCL) and the Behavior Rating Inventory of Executive Function (BRIEF) and academic information sourced from the BRIEF, CBCL, medical folders, and caregivers. Analyses include descriptive statistics and bivariate correlation matrices.

**Results:** The descriptive results show that (1) more than half of the participants experienced clinically-significant behavioral problems across the CBCL scales, (2) the working memory BRIEF subscale appeared to be the most problematic subdomain, (3) two thirds of the sample were receiving some form of, or were in the process of being placed in, special needs education, (4) there was a three-fold increase in the use of special education services from pre- to post-injury, and (5) more than half (*n* = 16) of the sample repeated at least one grade after returning to school post-injury. Correlation analyses results suggest that children with increased externalizing behavioral problems and executive dysfunction are more likely to repeat a grade post-injury; and that children with executive dysfunction post-TBI are more likely to require some form of special educational services.

**Conclusion:** While there is a vast amount of literature on pediatric TBI (pTBI) academic and behavioral outcomes, little literature exists on the pTBI population from the developing world and SA specifically. This is important to address given unique challenges that face the country and its educational system, and its implications for the management and care of children post-TBI.

## Introduction

### TBIs in developing world countries like South Africa (SA)

A disproportionate number of individuals who sustain traumatic brain injuries (TBIs) in the state sector are children and adolescents (Thurman, 2016). Although TBI is a global problem, and reported as a leading cause of mortality and morbidity among youth in high-income countries (HICs) the burden thereof is reportedly more potent in resource-poor countries (Hyder et al., 2007; Harris et al., 2008; Alexander et al., 2009; Kumar and Mahapatra, 2009; Bener et al., 2010; Figaji, 2017). This disproportionate effect on poorer economies is attributed to the quality of the environment and lack of resources. There are several factors often associated with resource-poor countries, such as poverty, lack of access to education, differences in infrastructure, and social problems, like alcoholism and higher road traffic accidents rates, that create greater risk for TBI (Levin, 2004; Hyder et al., 2007; Alexander et al., 2009). Further, home and work environments in indigent areas are often less secure, with residents more exposed to potential hazards. Prevention endeavors and access to rehabilitation may also be less available in such environments (World Health Organization, 2011). Other adverse factors that can impact on the incidence and/or consequences of TBI include the inconsistent scope of care for TBI survivors and health care facilities that are ill-prepared to cope with the degree of injury and care required for a public health problem of this magnitude (Hyder et al., 2007; Jerome et al., 2017). Hence, the burden of trauma, and in particular TBI, as the leading cause of death and neurological disability in trauma patients, is far greater in resource-poor countries than in the developed world (De Silva et al., 2009; Figaji, 2017).

The formidable economic sequelae associated with TBI results not only from the expenses associated with direct healthcare, but also from indirect costs linked to loss of the potential future productivity of that individual (Jaffe et al., 1993; Ragnarsson, 2002; Flanagan et al., 2008). The loss of potential future productivity is especially important in the case of children, clearly because most of their life will be spent in the shadow of the TBI. Also, there are associated costs, such as a loss of productivity for extended periods of time for caregivers. TBI can therefore be economically challenging and exhausting at the societal, individual and familial levels (Jaffe et al., 1993; Tilford et al., 2005; Gontkovsky et al., 2006). These factors are compounded in resource-poor environments.

Published literature on the epidemiology of pediatric TBIs (pTBIs) in developing world countries is generally limited. Researchers highlight the dearth of much-needed research of this nature generally and in SA specifically (Bruns and Hauser, 2003; De Silva et al., 2009; Haaring et al., 2011; Naidoo, 2013). Although it is suggested that the incidence of pTBI in SA must be high, exact rates are not available because systematic research on the topic is lacking (Levin, 2004; Penn et al., 2009; Corrigan et al., 2010; Naidoo, 2013; Tabish and Syed, 2015). There is little quantification of the neurological disability of survivors and the impact this has on families and state sector services.

### Education and inequality in SA

Although SA is considered a developing country with upper to middle income levels (The World Bank, 2016), its Gini index, which represents inequality in the spread of income, is among the highest in the world. This unequal socio-economic climate, which stems from the country's apartheid history, is also clear in the school system where inequality (in terms of financial and resource provision) is rife (Du Plessis, 2001; Engelbrecht, 2006; Soudien and Baxen, 2006; Donohue and Bornman, 2014). The apartheid-based Bantu Education Act of 1953 engendered such unequal education (Donohue and Bornman, 2014; Letgotlo, 2014). Even after SA's movement to a democracy in 1994, the scars of fragmentation from past segregation and discrimination practices remain, which has long resulted in the deprivation of adequate education for large numbers of SA people (Du Plessis, 2001; Engelbrecht, 2006).

Even though some progress has been made with the SA Schools Act (SASA) of 1996 to democratize and make uniform the school system post-democracy, and despite the knowledge that all learners (as per the Bill of Rights) have the right to equity and quality education, SA's schooling system is in a precarious state (Letgotlo, 2014, South African Council for Educators SACE, 2016). A recent news publication (The Economist, 2017) described some of the inequality (schools “with cricket pitched as smooth as croquet lawns” vs. others built from mud) and consequent dire outcomes (worrying percentages of learners who do not finish^1^ school and not being able to read or work out basic division sums after 5–6 years of schooling) associated with some of the country's schools.

There are also reported problems with schools for learners with special education needs (LSEN^2^) in the country. Donohue and Bornman (2014) report that as many as 70% of LSEN who should be in school given their age, are not; most of those who *are* in school attend LSEN, rather than mainstream schools. This finding contrasts with the inclusive^3^ education policy that the Department of Education (DOE) in SA have been aiming to implement, in line with the global trend of inclusive education and the Education for All initiative [United Nations Educational, Scientific, and Cultural Organization (UNESCO, 1990)]. SA's unique complex sociopolitical and economic background distinguishes it from other countries following this trend, however.

Amollo (2008) reports on a briefing by the SA DOE and their aims to reserve LSEN schools for those with severe disabilities and that as far as possible, have mainstream schools accommodate those with less severe disabilities, in a move from an exclusive toward a more inclusive educational model (Du Plessis, 2001). While a DOE policy document “White Paper 6” (Department of Education, 2001) was meant to frame and direct this transformation (Pillay and Di Terlizzi, 2009) toward inclusive education, the implementation thereof has been challenging and consequently poor (Engelbrecht, 2006; Donohue and Bornman, 2014).

In trying to understand the barriers to the implementation of inclusive education in the country, both societal and contextual factors need to be considered (Engelbrecht, 2006). Donohue and Bornman (2014) describe school-related and culture- and psychosocial-related barriers. In terms of the former, the authors conjecture that because most of the country's teaching complement are of the “older generation”, that some might not have embraced or repositioned their thinking (perhaps as a function of previous training) to align with the new inclusive education strategy. They do however note that low resources remain the main obstruction, even among those who are aligned with the new inclusive policy. Regarding culture- and psychosocial-related barriers, in some communities, individuals with disabilities may be devalued and prejudiced such that schooling may not be viewed as major priority for them, as their potential is questioned. On the other hand, parents may want to protect their children with special needs from ill treatment and stigma, and may therefore choose to keep them home.

Other authors question the preparedness of the country to promote inclusive education. Pillay and Di Terlizzi (2009) note that more resources and infrastructure (facilities) are needed in mainstream schools first. From their case study, they report that despite the move toward inclusive education, LSEN schools, though few, may still be better equipped to accommodate learners with special needs than mainstream schools. According to principals of mainstream schools, some of the main challenges to inclusive quality education, is educators coping with learners with special needs who are included in mainstream schooling, managing behaviorally challenged learners and behaviors and emotions of children who fail to progress, perceived lack of support (including parental support) and training for teachers and management. Large class sizes and considerable workloads for educators were some additional barriers reported for educators (Materechera, 2014).

SA DOE audits reported on by Amollo (2008) also revealed a weak infrastructure and a host of similar and other problems in terms of some LSEN schools locally including classroom overcrowding, ill-prepared educators, and lack of necessary assistive devices. Issues with learner progression and skill recognition, nutrition, transport, and discrimination were also listed. There were also challenges in terms of the lack of LSEN schools in rural areas and closure of several LSEN schools (Amollo, 2008; Materechera, 2014). Further, principals of LSEN schools perceive lack of funding, adequately trained staff and specialists, parental support, resources, and support services, as well as stigmatization of special needs school learners by mainstream school learners and acceptance by peers, diversity in disabilities and closure of special needs schools to be the main barriers to inclusive education (Materechera, 2014).

When one considers the experience of pTBI survivors in SA, the commonly reported academic and behavioral post-TBI challenges may be exacerbated by these contextual limitations of the burden of disease and barriers to accessing appropriate education.

### TBI outcomes

#### Academic outcomes

It is well-established globally that pTBI survivors experience impairments in a range of neurocognitive and behavioral domains, which may have adverse effects on academic outcomes (Van't Hooft, 2010; Li and Liu, 2012; Babikian et al., 2015). Changes in both academic performance and behavior can interfere with school re-entry, educational progress, and ultimately, quality of life of the injured child. These post-pTBI effects extend beyond the child to their familial and social environment (Donders, 1994; Anderson and Yeates, 2010; Treble-Barna et al., 2016).

Post-pTBI cognitive sequelae include deficits in general intellectual functioning, attention, executive function, memory and learning, and language skills (Rao and Lyketsos, 2000; Mayfield and Homack, 2005; Anderson and Yeates, 2010; Yeates, 2010; Babikian et al., 2015). Academic performance depends on the integrity of these cognitive skills. For example, a child's ability to sustain their attention, learn the material presented to them and then remember it, is pertinent to successful academic progression (Arroyos-Jurado et al., 2000; Hawley, 2004; Prasad et al., 2017). Executive functions (such as working memory and inhibition)—involved in the coordination of goal-directed behavior—also play an important role in academic achievement, as well as behavioral and adaptive functioning (Anderson et al., 2002; St Clair-Thompson and Gathercole, 2006). TBI can also affect core academic skills such as reading, writing, mathematics, and spelling; however, some academic skills may be more affected than others (Taylor, 2010). Further, the often-extended absence from school during the post-injury convalescent period, which can result in less opportunity for learning, can also contribute to poor academic outcome (Ewing-Cobbs et al., 1998; Babikian and Asarnow, 2009).

Knowledge of pTBI survivors and any cognitive deficits they may experience within a classroom is important because teachers may assume that pTBI survivors are fully recovered from their injuries when no obvious physical deficits are seen and some teachers may be unaware that learners in their classroom may have sustained a TBI, particularly when the injury occurred prior to the child entering that class (Hawley, 2004; Jantz and Coulter, 2007). This may result in the lack of, or delayed implementation of, academic assistance and modifications in the classroom required by pTBI survivors (Mayfield and Homack, 2005; Jantz and Coulter, 2007).

#### Behavioral outcomes

While a child's outcome may be significantly impacted by post-TBI cognitive impairment, it is often the behavioral changes that are considered more debilitating, particularly following severe TBI, given the dose-response relationship between severity and outcome (Donders, 1994; Babikian and Asarnow, 2009; Taylor, 2010; Catroppa et al., 2012). Behavioral problems not only interfere with the functioning and educational progress of the injured child, but can also be disruptive to others in the home, community, or classroom, particularly when these problem behaviors persist over time (Savage et al., 2005; Yeates and Taylor, 2006). Behavioral impairments negatively impact school performance, by hindering both the continued development of current skills and acquisition of new skills (Keenan and Bratton, 2006; Jonsson et al., 2013; Babikian et al., 2015).

Patterns of behavioral problems following TBI may vary from one child to another and may include *internalizing impairments* such as anxiety, withdrawal, depression, and other emotional problems and *externalizing impairments* such as aggression, irritability, disinhibition, impulsivity, agitation, and distractibility (Fletcher et al., 1990; Mayfield and Homack, 2005; Yeates and Taylor, 2006; Li and Liu, 2012). These behavioral impairments may occur as a direct result of damage to the brain and resulting cognitive deficits. For example, damage to the vulnerable frontal and associated areas may lead to impaired executive functioning, including the inability to initiate tasks, self-monitor behavior, and inhibit responses (Mayfield and Homack, 2005; Yeates, 2009). Problems with behavioral inhibition may lead to a hostile environment in the classroom, especially when these problems are expressed through agitation or inappropriate (e.g., insulting) comments or actions (e.g., getting out of one's chair), which can be disruptive to other learners in the classroom (Mayfield and Homack, 2005). Behavioral dysfunction can also be an indirect consequence of the injury (Mayfield and Homack, 2005). For example, children may react negatively and act out in trying to resume daily activities while adjusting to post-injury deficits, leading to frustration when previously managed tasks become more challenging. This may emerge as children become more aware of their deficits. Negative behaviors post-injury may also occur in response to the family's reaction to the injury. Parental stress and unrealistic expectations (especially in the absence of physical injuries) may also indirectly lead to behavioral impairments (Max et al., 1999; Donders and Strom, 2000; Taylor et al., 2001; Bamdad et al., 2003; Mayfield and Homack, 2005). The demands placed on the child may then lead to increased feelings of frustration and inadequacy; such undesirable feelings consequentially reinforce poor behavior (Kinsella et al., 1999; Savage et al., 2005; Li and Liu, 2012).

Changes in academic performance and impairments in behavior can interfere with educational progress and quality of life of the injured child. The consequences of TBI can also be burdensome to the child's family and others in their social and classroom environments (Anderson and Yeates, 2010; Li and Liu, 2012). These post-TBI sequelae are exacerbated within the context of a resource-poor country like SA where the education system is in a somewhat precarious state especially for those from lower socioeconomic backgrounds. While there is a vast amount of literature on pTBI academic and behavioral outcomes, little literature exists on the pTBI population from the developing world and SA specifically. This is important to address given unique challenges that face the country and its educational system, and its implications for the management and care of children post-TBI. The purpose of this cross-sectional, descriptive study was therefore to investigate and describe behavioral and academic outcomes following severe TBI in a group of South African children of school-going age.

## Methods

### Sample

The sample included 27 children with severe TBI who were of school-going age at the time of their injury. These children were identified from a database of 137 children who had been admitted to Red Cross War Memorial Children's Hospital (RXH) for severe TBI (post-resuscitation Glasgow Coma Scale (GCS)^4^ score of ≤ 8) over a 5-year period (2006–2011) and who underwent intracranial monitoring (Schrieff et al., 2013).

**Figure 1:**
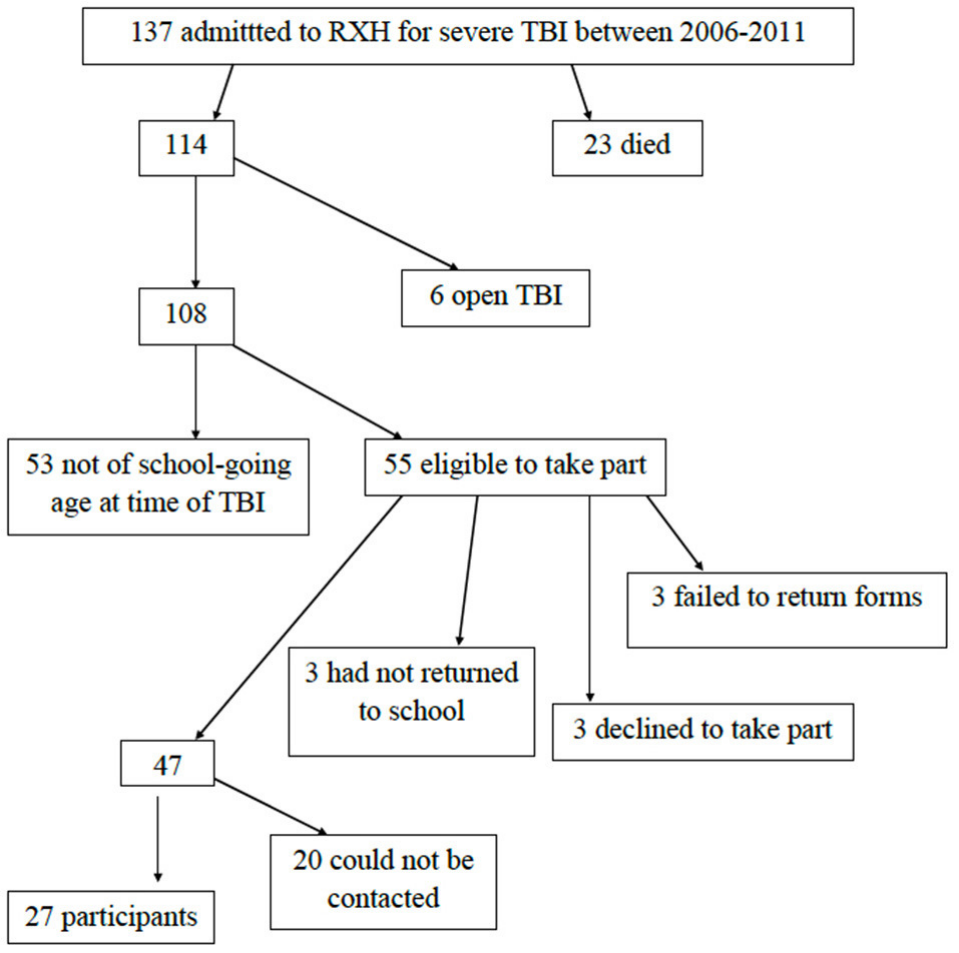
Flowchart of how the sample of 27 participants was obtained.

Participants had to be at least 1 year post-injury, because the recovery trajectory reportedly then tends to stabilize (Jaffe et al., 1995; Taylor, 2010). We excluded children who had sustained open TBIs, given the differing pathophysiology and outcomes (compared to closed TBI; Anderson et al., 2001), and those who were not attending school at the time of injury and assessment, as this limited the academic data available for those children. Figure 1 shows the reasons for exclusion of participants, resulting in a final sample of 27 children.

### Procedure

We contacted caregivers of potential participants telephonically or approached them at their follow up Neurosurgery outpatient clinic visit if contact details were unavailable in the medical folders^5^. We collected the data between 2012 and 2013. Three caregivers completed the measures at their homes due to time or transport constraints; the remainder at RXH. First language isiXhosa-speaking research assistants assisted with interpreting when necessary.

### Measures

We had the original English versions of our measures translated into two other common local languages, Afrikaans and isiXhosa, to facilitate administration. These were linguistically validated through forward and back translations and authentication by Stellenbosch University's Language Services (Cape Town, SA).

We used a parent information questionnaire and asset index (Myer et al., 2008) to obtain demographic and socioeconomic background information of the participants. It captures demographic information such as parental/guardian employment and education, home language, and annual household income. The asset index groups asset ownership into three categories: 0–5 (low), 6–12 (medium), and 13–17 (high) and reflects the material and financial resources of the household, for example, appliances (e.g., microwave oven, refrigerator, television), a flushing toilet, running water and car, as well as whether the responder makes use of bank accounts and credit cards.

We used a questionnaire from the RXH pediatric neuropsychology clinic to obtain information on the developmental history (including pregnancy and birth, development, and family composition) of the participants.

We used the informant (parent) measures of the Child Behavior Checklist (CBCL; Achenbach and Rescorla, 2001) and the Behavior Rating Inventory of Executive Function (BRIEF; Gioia et al., 2000) to assess behavioral and emotional functioning, and executive functioning, respectively. These are recognized ecologically valid tools for assessing these outcomes (Schwartz et al., 2003; Gioia and Isquith, 2004).

The CBCL is a 112-item questionnaire used to assess behavioral and emotional functioning of children aged 6–18 years (Achenbach and Rescorla, 2001). Besides demographic questions, there are items that assess competency of activities, social functioning and school. Scores are also produced for eight syndrome scales: Anxious/Depressed, Withdrawn/Depressed, Somatic Complaints, Social Problems, Attention Problems, Rule-breaking Behavior and Aggressive Behavior, and six Diagnostic and Statistical Manual (DSM)-oriented scales: Affective Problems, Anxiety Problems, Somatic Problems, Attention Deficit/Hyperactivity Problems, Oppositional Defiant Problems, and Conduct Problems. On the syndrome and DSM-Oriented scales, age-standardized T-scores above 70 suggest clinically significant behavioral problems, and those from 67 to 70 are borderline clinical. Scores are also produced for two broad syndrome groups: Internalizing Problems and Externalizing Problems that together with the remaining syndrome scales produce a Total Problems score. Age-standardized T-scores above 63 suggest clinically significant behavioral problems and those from 60 to 63, are borderline clinical. The CBCL has both reported validity and reliability (Achenbach and Rescorla, 2001). There are some published research studies using the CBCL with South African samples (e.g., Shields et al., 2008; Palin et al., 2009; Schrieff-Elson et al., 2015).

The BRIEF is an 86-item standardized rating scale assessing everyday executive function behaviors within the home environment for children aged 5–18 years (Gioia et al., 2000). Two index scores [Behavior Regulation Index and Metacognition Index] and an overall composite score of executive function, the Global Executive Composite (GEC), are produced. Scores for eight clinical subscales, that assess interrelated executive function domains, are also produced. Inhibit, Shift, and Emotional Control are subscales of the BRI, while Initiate, Working Memory, Plan/Organize, Organization of Materials, and Monitor are subscales of the MI. T-scores of ≥63 are clinically relevant. The BRIEF shows high levels of internal consistency and stability, as well as test-retest reliability (Malloy and Grace, 2005; Chapman et al., 2010). It has been used cross-culturally in published studies, for example, in the Han Chinese and Dutch populations (Qian et al., 2010; Huizinga and Smidts, 2011) and in a published SA study (Schrieff-Elson et al., 2015).

We sourced academic information regarding education type and grade repetition for the entire sample from relevant questions on the CBCL. For four participants, *specific details* of the academic data (i.e., whether the child was making use of remedial services or not) were unclear; we therefore consulted the caregiver directly and/or consulted the participants' case files for these details.

### Statistical analysis

We used SPSS 22.0 to compute the analyses.

#### Descriptive statistics

We used descriptive statistics to present the demographic and clinical characteristics of the participants.

#### Dependent variables

##### Academic outcomes

Academic outcome is represented by two variables: (1) the child's education type at the time of assessment, and (2) whether the child had repeated any grades since returning to school after their injury (see Table 1 for subcategories). The latter encompasses whether the child had to repeat the grade that they were in at the time of their injury (due to prolonged absence or for academic reasons), or whether they had to repeat subsequent grades post-injury. Both the education type and grade repetition variables were assigned values and converted to *z*-scores for purposes of statistical analyses. Greater scores represent poorer academic outcome on each of the variables.

**Table 1:** Variables that represent academic outcome as assessed in this sample.

**Variable**	**Value**
Education type at time of assessment	0 mainstream school
	1 mainstream school with remedial help
	2 mainstream school with special needs school application or awaiting placement
	3 special needs school
Repeated grades since returning to school	0 no repeated grades since returning to school
	1 repeated a grade since returning to school

##### Behavioral outcomes

For correlational analyses, we used the scores from the CBCL Total, Internalizing, and Externalizing Problems scales, and the BRIEF's BRI, the MI, and the GEC scores. Subscale scores from the CBCL and index scores from the BRIEF formed part of the descriptive statistics. Greater scores represent more problem behaviors on both the CBCL and BRIEF.

#### Correlation matrices

We used bivariate correlation matrices (Pearson and Spearman's correlation coefficients) to explore the relationships between the academic and behavioral outcome variables. We used one-tailed correlational analyses based on established literature, which informed the direction of the expected relationships. The *r*-statistic provided a measure of effect size which are described as small, medium or large and represented by *r*-values of 0.10, 0.30, and 0.50, respectively (Field, 2009).

## Ethical considerations

This protocol was approved by the University of Cape Town (UCT)'s Department of Psychology's Research Ethics Committee and Faculty of Health Sciences' Human Research Ethics Committee (Ref: 345/2011). We obtained written informed consent, as well as permission to obtain school data, from the caregivers of all children in the sample. All data were obtained from parents or legal guardians; hence we did not obtain assent as the children themselves did not complete any measures. This study adhered to the World Medical Association Declaration of Helsinki's ethical principles for medical research involving human subjects (World Medical Association, 2001). We obtained permission from the Western Cape Education Department, SA, to access school data.

## Results

Tables 2, 3 present a description of the demographic and SES, and the injury characteristics of the sample, respectively. As per Table 2, more than half of the participants were isiXhosa-speaking and male. All participants had access to at least six or more material and financial resources (medium or high asset index bracket). These do, however, include basic amenities as previously described. Half of the caregivers of the children in the sample earned up to ZAR25 000 per annum, with 37% reporting earnings from ZAR25 000 to ZAR100 000 per year. This latter result, however, includes quite a wide range and one cannot be sure how many families are earning closer to the lower end of that range. Most parents had 8–11 years education. The youngest participant was 6 years 5 months, while the oldest was 12 years 7 months at the *time of injury*. The range of time since injury was 15 to 70 months (Table 3).

**Table 2:** Demographic and SES characteristics of the sample (*N* = 27).

**Variable**	**Frequency (%)**
**SEX**	
Male: female	17: 10 (62.96: 37.04)
**AGE IN MONTHS AT ASSESSMENT**	
Mean (*SD*)	151 (24.00)
Range	115–195
**HOME LANGUAGE**	
English	5 (18.52)
Afrikaans	5 (18.52)
English and Afrikaans	2 (7.41)
isiXhosa	14 (51.85)
Other^a^	1 (3.70)
**ANNUAL HOUSEHOLD INCOME**^b^	
0	1 (3.70)
1–5,000	6 (22.22)
5,001–25,000	8 (29.63)
25,000–100,000	10 (37.04)
100,001+	2 (7.41)
**PARENTAL EDUCATION (FATHER: MOTHER: GUARDIAN)**^c^	
0 years	1: 0: 0
1–6 years	0: 0: 0
7 years	5: 3: 0
8–11 years	10: 12: 1
12 years	3: 7: 0
13+ years	3: 3: 0
Unknown/incomplete	4: 1: 0
**MATERIAL AND FINANCIAL RESOURCES (ASSET INDEX)**	
0–5 assets (low)	0 (0)
6–12 assets (medium)	15 (55.55)
13–17 assets (high)	12 (44.44)

a *The home language recorded as being “other” was Swahili. However, the caregiver was fluent in English and therefore the participant was not excluded*.

b *Household income presented in South African Rands (ZAR)*.

c *For one of the cases, information on education and employment was only provided for the participant's guardian and not for the parents*.

**Table 3:** Injury characteristics of sample (*N* = 27).

**Variable**	**Mean (*SD*)**
**AGE AT INJURY (MONTHS)**	
Mean (*SD*)	114.11 (21.94)
Range	78–152
**TIME SINCE INJURY (MONTHS)**	
Mean (*SD*)	37.22 (15.19)
Range	15–70
**GCS**	
Mean (*SD*)	5.03 (1.56)
Range	3–8

*Means are presented with standard deviations in parentheses. GCS, Glasgow Coma Scale*.

### Academic outcome

Table 4 compares the academic information obtained for the sample and presents the ratios of grade repetition, and type of education pre- and post-injury. When considering the enrollment in remedial and special needs education services from pre- to post-injury, there was a three-fold increase from before (*n* = 6; 22.22%) to after (*n* = 18; 66.67%) the TBI. This increase reveals that for 44.44% of the sample (*n* = 12), the use of remedial services or special needs education was required only after the TBI. Remedial help was usually in the form of extra lessons or placement in a remedial-oriented class at the mainstream school. For six of these 12 participants, school personnel were in the process of applying to a school that catered for their specific learning or physical needs and were awaiting the outcome of this application, or applications were already approved and they were awaiting transfer. In these cases, children continued to attend mainstream schools in the interim, where they may or may not have received remedial help during that time. Four (14.81%) of children in special needs schools at the time of the study had been placed in those schools when they resumed schooling post-injury, or were placed a later stage before commencement of the study. More than half of the sample (*n* = 16; 59.26%) had repeated a grade following their injury, which represents a 275% increase from pre- to post-TBI.

**Table 4:** Academic information obtained for the sample (*N* = 27).

**Component**	**Pre-injury frequency (%)**	**Post-injury frequency (%)**
Type of education		
Mainstream	21 (77.78)	9 (33.33)
Mainstream with remedial help	5 (18.52)	7 (25.93)
Mainstream with special needs	0 (0)	6 (22.22)
application in place or awaiting placement		
Special needs education	1 (3.70)	5 (18.52)
Repeated grade: Did not repeat grade	4:23 (14.81: 85.19)	16:11 (59.26: 40.74)

*Frequencies are presented with percentages in parentheses*.

**Figure 2:**
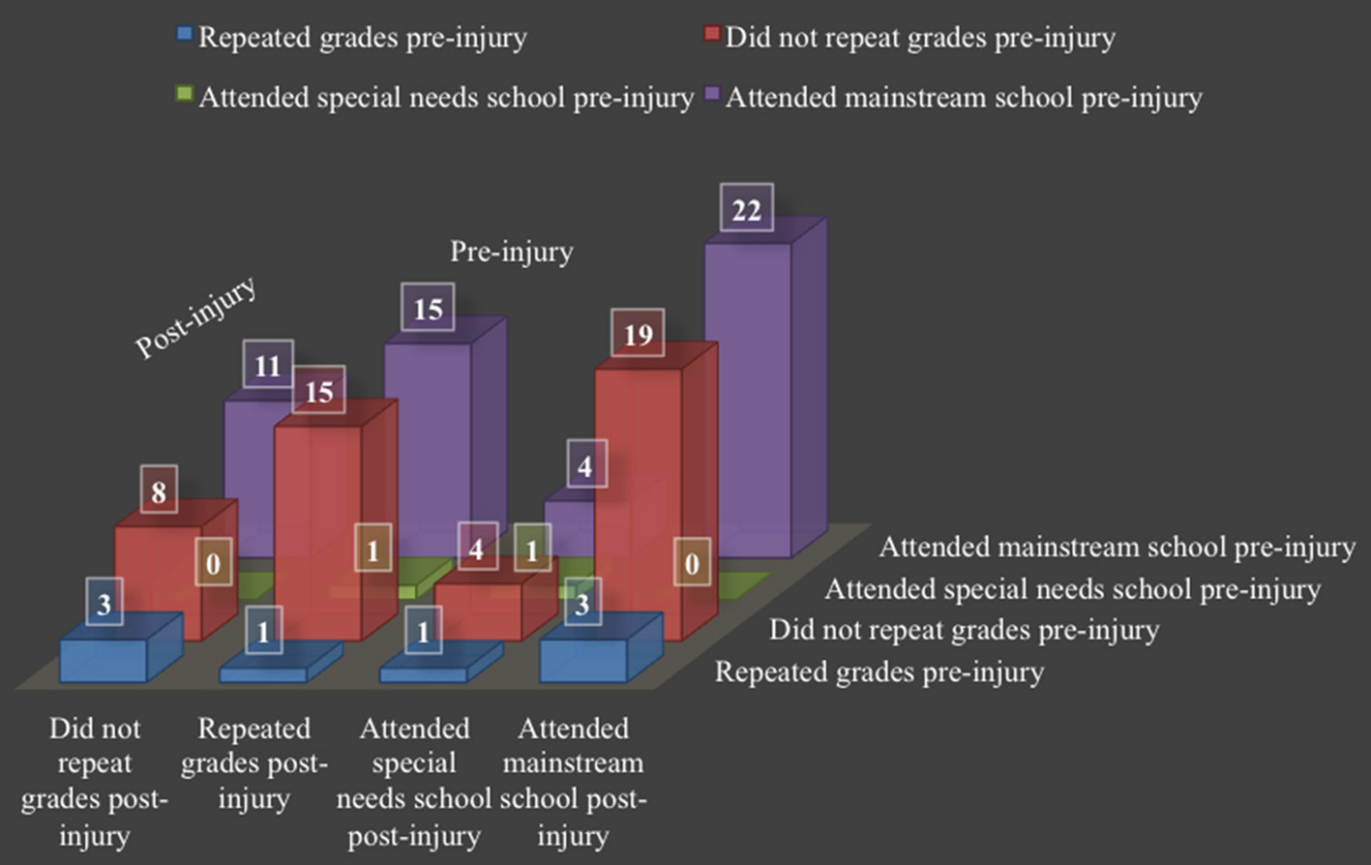
Cross-tabulation of pre- and post-injury academic information.

Figure 2 presents a cross-tabulation of pre- and post-injury academic information. In the sample, 15 (55.55%) children who had not repeated a grade pre-injury, went on to repeat one or more grades post-injury. Of the four participants (14.80%) that repeated a grade pre-injury, only one (3.70%) also repeated a grade post-injury. This participant attended a special needs school pre- and post-injury and had premorbid cerebral palsy. The three participants (11.10%) who repeated a grade pre-, but not post-injury, attended mainstream schools both pre- and post-injury. Only one of these participants required the use of remedial services post-injury. Of those that attended mainstream school pre-injury (*n* = 26; 96.30%), four (14.81%) were then placed in special needs school post-injury.

### Behavioral outcomes

#### CBCL

Table 5 presents the descriptive statistics for the CBCL scores. Caregivers reported clinically significant Total Problems (65.38%), Internalizing Problems (65.38%), and Externalizing Problems (53.85%) for more than half of the participants. Although fewer participants scored within the clinical range on all other individual CBCL scales and subscales, some of these frequencies were still above 40% [e.g., Aggressive Behavior (46.15%) and Affective Problems (46.15%) scales].

**Table 5:** Outcome scores obtained on the CBCL parent version (*N* = 26).

**CBCL Syndrome Profile**	**Mean (*SD*)**	**Range**	**Frequency of borderline clinical scores (%)**	**Frequency of clinical scores (%)**
Total problems	68.50 (9.85)^a^	47–88	3 (11.54)	17 (65.38)
Social problems	67.77 (13.57)^b^	50–100	6 (23.08)	8 (30.77)
Thought problems	63.72 (11.25)	50–90	5 (19.23)	8 (30.77)
Attention problems	68.42 (11.87)^b^	51–100	3 (11.54)	10 (38.46)
Internalizing problems	67.27 (9.87)^a^	48–89	4 (15.38)	17 (65.38)
Anxious/depressed	65.31 (10.57)	50–94	3 (11.54)	8 (30.77)
Withdrawn/depressed	65.23 (10.20)	50–93	4 (15.38)	5 (19.23)
Somatic complaints	66.08 (10.07)	50–90	8 (30.77)	7 (26.92)
Externalizing problems	66.19 (11.92)^a^	46–88	4 (15.38)	14 (53.85)
Rule-breaking behavior	63.12 (11.22)	50–86	4 (15.38)	7 (26.92)
Aggressive behavior	69.58 (13.94)^b^	50–100	1 (3.85)	12 (46.15)
**DSM-ORIENTED SCALE**				
Affective Problems	68.58 (9.64)^b^	50–87	1 (3.85)	12 (46.15)
Anxiety Problems	60.58 (8.51)	50–100	3 (11.54)	4 (15.38)
Somatic Problems	64.65 (9.93)	50–93	4 (15.38)	5 (19.23)
ADH Problems	65.96 (8.07)	52–80	6 (23.08)	6 (23.08)
Oppositional Defiant Problems	63.46 (10.74)	50–80	3 (11.54)	9 (34.62)
Conduct Problems	67.00 (12.63)^b^	50–93	5 (19.23)	8 (30.77)

*Means are presented with standard deviations in parentheses. Frequencies are presented with percentages in parentheses. One participant's scores are missing from the CBCL. The caregiver did not correctly complete the form and it could not be scored. The caregiver subsequently moved away and was lost to follow-up. CBCL, Child Behavior Checklist; ADH, Attention Deficit/Hyperactivity*.

a *Clinical range*.

b *Borderline clinical range*.

#### BRIEF

Table 6 shows that on the BRIEF, the mean reported scores for most of the indices fell within the clinical range, with Working Memory Index (73.08%) as the highest, followed by Emotional Control (65.38%), and Plan/Organization (65.38%). Overall on the GEC, 69.23% of the sample scored in the clinical range. Looking specifically at the major index scales, more than half the sample scored within the clinical range on the BRI (61.54%) and the MI (61.54%).

**Table 6:** Outcome scores obtained on the BRIEF parent version (*N* = 26).

**Indices**	**Mean (*SD*)**	**Range**	**Frequency of clinical scores (%)**
GEC	69.19 (14.58)^a^	47–97	18 (69.23)
BRI	70.65 (17.47)^a^	45–101	16 (61.54)
Inhibit	67.62 (18.82)^a^	40–103	14 (53.85)
Shift	68.54 (15.85)^a^	40–98	14 (53.85)
Emotional control	68.04 (14.97)^a^	43–91	17 (65.38)
MI	66.08 (12.07)^a^	45–87	16 (61.54)
Initiate	61.77 (11.88)	43–89	10 (38.46)
Working memory	70.00 (10.00)^a^	50–87	19 (73.08)
Plan/organization	64.46 (11.08)^a^	45–88	17 (65.38)
Organization of materials	58.08 (9.85)	45–72	9 (34.62)
Monitor	63.35 (13.78)^a^	40–86	15 (57.69)

*Means are presented with standard deviations in parentheses. Frequencies are presented with percentages in parentheses. Data was missing for one participant. The caregiver of this participant had taken the measures home with him to complete; all measures were returned apart from the BRIEF. He was subsequently lost to follow-up due to a change in contact details after changing employment. BRIEF, Behavior Rating Inventory of Executive Function; BRI, Behavior Regulation Index; MI, Metacognition Index; GEC, Global Executive Composite*.

a *Clinical range*.

### Correlation matrix

Table 7 shows the correlations between academic and behavioral outcomes. There were strong significant positive correlations between repeated grade post-injury and the CBCL Total Problems and CBCL Externalizing Problem scales and the three BRIEF indices. These relationships suggest that grade repetition post-injury was associated with poorer reported scores on these behavioral measures.

**Table 7:** Correlation matrix for academic outcome and behavioral outcome variables (*N* = 27).

	**Variable**	**1^a^**	**2^a^**	**3^b^**	**4^b^**	**5^b^**	**6^b^**	**7^b^**	**8^b^**
1.	Repeated grade post-injury^a^	–	–	–	–	–	–	–	–
2.	Post-injury school type^a^	0.271	–	–	–	–	–	–	–
3.	CBCL Total Problems^b^	0.471^**^	0.182	–	–	–	–	–	–
4.	CBCL Internalizing Problems^b^	0.195	−0.67	0.873^**^	–	–	–	–	–
5.	CBCL Externalizing Problems^b^	0.528^**^	0.193	0.933^**^	0.800^**^	–	–	–	–
6.	BRIEF GEC^b^	0.602^**^	0.413^*^	0.768^**^	0.455^*^	0.813^**^	–	–	–
7.	BRIEF BRI^b^	0.565^**^	0.345^*^	0.819^**^	0.557^**^	0.861^**^	0.945^**^	–	–
8.	BRIEF MI^b^	0.597^**^	0.488^**^	0.672^**^	0.345^*^	0.727^**^	0.962^**^	0.822^**^	–

*CBCL, Child Behavior Checklist; BRIEF, Behavior Rating Inventory of Executive Function; BRI, Behavior Regulation Index; MI, Metacognition Index; GEC, Global Executive Composite; Statistics presented are Pearson correlation coefficients (r) unless otherwise stated. All tests are 1-tailed*.

a *Statistics presented are Spearman correlation coefficients for continuous variables that are not normally distributed (r_s_)*.

b *n = 26*.

* p < 0.05.

** *p < 0.01*.

There were medium to strong positive correlations between post-injury school type and the BRIEF indices, suggesting that poorer executive function behaviors were associated with increased need for remedial and special needs education.

As expected, were the significant positive correlations between outcome scores on the CBCL and the BRIEF, which were all in the expected direction.

## Discussion

This study aimed to contribute to the pTBI outcome literature in a developing world setting, by investigating and describing behavioral and academic outcomes in a group of school-going South African children who had sustained a severe TBI.

### Academic outcomes

Two thirds (18/27) of the sample were, at the time of assessment, receiving some form of remedial or special needs education or were in the process of being placed in remedial or special needs education post TBI. Thus, there were more children requiring some form of specialized education following their TBI than those who were integrated back into mainstream schooling. This result is consistent with literature on pTBI survivors being reintegrated into the schooling system and the associated increased need for specialized educational services, particularly with more severe TBI (Donders, 1994; Kinsella et al., 1997; Ewing-Cobbs et al., 1998; Savage et al., 2005; Jantz and Coulter, 2007; Arnett et al., 2013; Prasad et al., 2017). Taylor et al. (2003) found that 62% of the children with severe TBI in their sample were in programs that catered for special education needs, even several years after their injuries. Placement in these programs occurred soon after injury. Kinsella et al. (1997) previously reported similar high rates, where 70% of children with severe TBI required special needs intervention or attended school part-time after their injury; while Donders (1994) reported a 40% increase in the number of children requiring special needs education and Hawley (2004) a two-fold increase in the number children in her TBI (mixed severity) sample. In the current study, there was a three-fold increase (from *n* = 6 to *n* = 18) in the number of children using some form of special education services, from pre- to post-TBI.

Post-injury, 22% (6/27) of learners had applied for placement in a LSEN school, or had already been offered placement, but were awaiting transfer to that school. There are several reasons that could account for the delay in placement at an LSEN school. It may reflect a delayed process of identification of needs, for example, when deficits are not immediately evident and but are only evident at a later stage when cognitive demands on the child increase. The delay in placement may also be due to the unavailability of a place at a suitable school, especially when resources are limited (Taylor et al., 2003; Mayfield and Homack, 2005; Ciccia and Threats, 2015). In SA, the delay in placement in LSEN schools is often due to it being a lengthy bureaucratic process. Furthermore, the number of special needs schools as well as their capacity for learners is limited with more than 10,000 learners on a waiting list for placement (Amollo, 2008; Donohue and Bornman, 2014). This lengthy waiting list is particularly problematic when one considers that once learners reach 16 years of age, they are considered too old to be placed in LSEN schools.

More than half (59%) of the sample had to repeat at least one grade after returning to school post-injury. This result is consistent with the outcomes for the “children group” (5–10 years) in the Ewing-Cobbs et al. (1998) study with 55% having repeated a grade within two years following their injury. There was a four-fold increase in the number of children who had to repeat one or more grades from before to after the TBI.

The reported reasons for these adverse academic outcomes in the literature are varied. The cognitive deficits associated with TBI, including difficulties in attention, memory, executive function, and essential skills such reading and writing, can impact on academic performance (Donders, 1994; Ewing-Cobbs et al., 1998; Arroyos-Jurado et al., 2000; Hawley, 2004; Taylor, 2010; Max, 2014). Other factors such as behavioral impairments and absence from school may also play a role (Ewing-Cobbs et al., 1998; Babikian and Asarnow, 2009). In some cases, the child missed out on weeks or months of school during their post-TBI recovery period, especially when the TBI occurred during the school term. Frequently, the child also missed periods of school to attend doctors' appointments even after the initial hospital stay.

Given the already fragile state of the education system in SA and worrying statistics like 70% of children with special educational needs who should be in school, are not attending school, and given that many pTBI survivors (especially after severe TBI) have post-TBI special needs, one cannot help but be concerned about the future educational retention of these children. The tentative nature and lack of efficacy of the inclusive educational model also creates uncertainty around where SA pTBI survivors may be and are best placed.

### Behavioral outcome

#### CBCL

The sample's scores on the Total Problems, Internalizing Problems, and Externalizing Problems scales were on average in the clinically significant range, with relatively homogenous means. Elevated syndrome and DSM-Oriented scale scores, such as Aggressive Behavior, Affective Problems, Attention problems, Social Problems, and Conduct Problems, fell in the borderline clinical range. Overall, caregivers reported a greater frequency of clinically significant internalizing than externalizing problems. Such problem behaviors may persist over time and patterns thereof may vary. Within the internalizing and externalizing behavior scales, anxious/depressed behaviors and aggressive behaviors had the greatest frequency of clinically significant scores, respectively. The generally high occurrence of problem behaviors is consistent with numerous studies documenting the commonly reported sequelae following severe TBI and may reflect difficulties associated with self-regulation of behavior and emotions (see e.g., Fletcher et al., 1990; Kinsella et al., 1999; Mayfield and Homack, 2005; Yeates and Taylor, 2006; Dooley et al., 2008; Anderson and Yeates, 2010; Taylor, 2010; Babikian et al., 2015; Catroppa et al., 2015). Within both the internalizing and externalizing behavioral categories, although internalizing behaviors were generally more pronounced, on average, caregivers rated a greater degree (as indicated by the highest score) of aggressive behaviors than any other *specific* problem behavior assessed on the CBCL. While aggression can manifest as a direct result of damage to the brain and the associated cognitive deficits, Dooley et al. (2008) reported that aggressive behaviors are likely a result of anger and distress in response to one's injury and deficits, but may also be associated with emotional lability and decreased frustration tolerance.

#### BRIEF

In more than half of the sample, caregivers reported clinically significant problems across a range of executive function subdomains. Working memory appeared to be the most problematic. The results are consistent with literature documenting executive dysfunction in children who have sustained a TBI (Levin and Hanten, 2000; Anderson et al., 2002; Bamdad et al., 2003; Yeates et al., 2005; Yeates, 2010; Arnett et al., 2013). Working memory is crucial to optimal functioning of cognition and the assessment thereof generally (e.g., holding instructions in mind while executing a task) and is particularly susceptible to the effects of a TBI (Hillary et al., 2006). The high incidence of executive dysfunction, in addition to other problems with behavioral and emotional regulation (such as assessed on the CBCL and the BRIEF) in the sample likely reflect damage to the vulnerable frontal areas and associated neural circuitry mediating executive functioning and the regulation and self-monitoring of responses (Bamdad et al., 2003).

To promote inclusive education, the intention of the SA DOE is that only learners deemed to have severe disabilities be placed in LSEN schools, with learners with less severe disabilities being accommodated in mainstream schools (Amollo, 2008). However, it seems that educators may not all be adequately trained in this regard. Coping with learners with special needs in mainstream schools, managing behaviorally challenged learners (who could include pTBI survivors), and supporting those who fail to progress (who may struggle emotionally) are reported as concerns for educators and a barrier to the inclusive education model.

### Correlation analyses

The significant correlations between repeated grade post-injury and the Externalizing Problems scale (and Total Problems, which is likely a function of this) on the CBCL suggest that pTBI survivors who repeated a grade post-injury may display a greater the degree of externalizing behavior problems as assessed on the CBCL, or conversely, that those with externalizing problems, specifically showing more rule-breaking or aggressive behavior, are more likely to repeat grades after their injury. These findings are consistent with literature describing the impact of behavioral impairments on school performance and educational outcome (Keenan and Bratton, 2006; Babikian and Asarnow, 2009; Arnett et al., 2013). Nelson et al. (2004) found that among students who had emotional or behavioral disorders, those who exhibited externalizing as compared to internalizing behavioral problems were more likely to have deficits in academic achievement. These authors conjecture that externalizing behaviors (such as inattentiveness and disruptive behaviors) may have more of a pervasive influence than internalizing behavior problems with regards to interfering with the learning process and consequently coping and progressing academically. This is especially the case when the behaviors involve poor behavior regulation and self-monitoring secondary to executive dysfunction (Mayfield and Homack, 2005).

The positive significant associations between post-injury repeated grade and school type, and the outcome scores (GEC, BRI, MI) on the BRIEF, suggest that pTBI survivors with increased executive dysfunction, including problems with regulation of behavior and abilities related to problem solving, are more likely to be in need of special educational services or to have failed grades. The relationships between the academic outcome variables and the BRIEF outcomes are consistent with literature documenting the role of executive functions in the learning process and in academic achievement, as well as post-TBI executive function outcomes generally (Anderson et al., 2002; St Clair-Thompson and Gathercole, 2006; Babikian et al., 2015). In their sample of children and adolescents with Attention-Deficit Hyperactivity Disorder (ADHD), St Clair-Thompson and Gathercole (2006) found that there was increased risk of repeating grades, learning disabilities and poor academic achievement amongst those with deficits in executive functioning.

The significant positive relationships between the CBCL and BRIEF were expected due to the construct validity of these measures, and because the Internalizing Problems and Externalizing Problems scales, and the BRI and MI form part of the overall outcome scores (Total Problems and GEC, respectively) obtained on these measures.

In summary, the results show elevated problems with behavioral and executive functioning, and academic concerns in the sample. More than half of the participants experienced clinically significant behavioral problems and working memory appeared to be the most problematic subdomain of executive function. Two thirds of the children were receiving some form of, or in the process of being placed in, LSEN schools; and the increase in the number of children using some form of special education services from pre- to post-injury was three-fold. Furthermore, more than half of the sample repeated at least one grade after returning to school following their injury. The results in this study suggest that children with increased externalizing behavioral are more likely to repeat a grade post-injury; and that children with executive dysfunction post-TBI may be more likely to require some form of special educational services and more likely to repeat a grade post-injury (although the converse may also be true in both cases given the correlational nature of the study).

### Limitations and directions for future research

Ideally, the study design should include a model of change in academic grade performance and would incorporate a change in performance over three time periods: the period before injury, the initial period upon return to school, and the time of assessment. A change in performance could then be determined as a percentage recovery from the initial decline in academic performance and the total decline in academic performance. This model of change was however not achievable due to challenges in obtaining complete academic history (pre-injury scholastic and behavioral assessments, and term and year-end results) for most the sample. These limitations are not uncommon for a developing world setting and are important to address because it limits good data collection.

Due to the small sample size, the results of this study should be viewed with caution. The exclusion of children who were not attending school at the time of the study became a limiting factor in the size of the sample. Furthermore, the small sample had a wide age range of participants (6–12 years).

Further, this study made use of parent self-report measures, which may be limited by response sets, social desirability bias, and sometimes unreliable recall from memory of past behaviors. The measures used have not been validated in the SA context—as is the case generally with neuropsychological measures (Schrieff-Elson et al., 2017). Nevertheless, informant measures were used in this study due to their psychometric properties and that they are commonly used assessment tools. Moreover, they are more ecologically valid that standard pencil and paper measures, despite the lack of norms (Anderson et al., 2002; Gioia and Isquith, 2004). Further studies should look to supplement informant measures, for example, by including structured interviews with caregivers and teachers, as well as direct observations of behavior.

## Summary and conclusion

We investigated and described behavioral and academic outcomes for a group of school-aged severe pTBI survivors in SA. The results show that problems (e.g., increased need for special education services, behavioral problems and executive dysfunction) experienced by this sample are consistent with those reported for children with severe TBI in the literature (Babikian and Asarnow, 2009; Van't Hooft, 2010; Babikian et al., 2015; Prasad et al., 2017). The current study's results therefore advocate for increased awareness in identifying children, and indeed families, that are at greater risk for dysfunction and poorer academic outcomes following pTBI. This is particularly important in the developing world context like SA, where there are a limited number of LSEN schools (reserved for children with severe disabilities) and none that specifically cater for the unique needs of children with TBI (Levin, 2004). Interim remedial support should be provided until children who require special needs schooling are placed appropriately. For those who recover sufficiently to be accommodated in mainstream schools, ideally, this would include an increased availability of educational resources and learner support that focuses on the cognitive, behavioral and emotional sequelae associated with TBI. Advances in technology provide opportunities through which to view post-pTBI educational opportunities and support in developed compared to the developing world contexts and the obvious disparities in post-pTBI education reintegration. Clearly, changes in policy and a greater funding focus on this issue in the developing world context are needed (Chomba et al., 2014).

## Author contributions

AD carried out this research as her Masters research project. She was the lead author on for the write-up of this manuscript. AF was a co-supervisor on AD's MA research. He oversaw drafts of this article and provided edits and feedback. LS-E was the main supervisor for AD's MA research. She has overseen multiple drafts of the thesis and manuscript and provided edits and feedback.

### Conflict of interest statement

The authors declare that the research was conducted in the absence of any commercial or financial relationships that could be construed as a potential conflict of interest. The handling Editor declared a shared affiliation, though no other collaboration, with the authors.

## Footnotes

^1^Legotlo (2014) reports that 10% of learners complete school within a reasonable period of time.

^2^From Du Plessis (2001) based on the 1995 White paper: “The term ‘learners with special education needs’ refer to all learners in need of educational support, it is learners whose special needs arise from intrinsic factors as well as learners whose needs arise from extrinsic (social, structural and systematic) factors” (p. 62).

^3^“…a means of education according to which the learner is schooled in the least restrictive environment possible, to overcome his or her challenges to learning and development” (Pillay and Di Terlizzi, 2009, p. 491).

^4^The GCS has traditionally been used to classify severity of a TBI as mild, moderate or severe. Severe injury corresponds to a score of 8 or below on the GCS, moderate injury from 9 to 12, and mild injury a score of 13 or higher (Zillmer et al., 2008).

^5^Hereafter referred to as “case file;” while the hospital medical folder is primarily used to store medical information and documents, non-medical information such as demographics, correspondence and school results can also be found in it.
